# Inhibition of Akt Activity and Calcium Channel Function Coordinately Drive Cell-Cell Fusion in the BeWO Choriocarcinoma Placental Cell Line

**DOI:** 10.1371/journal.pone.0029353

**Published:** 2012-01-19

**Authors:** Manu Vatish, Lydia Tesfa, Dimitris Grammatopoulos, Eijiro Yamada, Claire C. Bastie, Jeffrey E. Pessin

**Affiliations:** 1 Division of Reproduction, Warwick Medical School, University of Warwick, Coventry, United Kingdom; 2 Department of Medicine and Molecular Pharmacology, Albert Einstein College of Medicine, Bronx, New York, United States of America; 3 Metabolic and Vascular Health, Warwick Medical School, University of Warwick, Coventry, United Kingdom; Roswell Park Cancer Institute, United States of America

## Abstract

To establish a simple and quantitative live cell fusion assay for placental syncytialization, we generated stable GFP and dsRed expressing fusogenic BeWo cell lines. Fluorescent Activated Cell Sorting was shown to provide a quantitative determination of forskolin (cAMP-mediated) fusion in a time and concentration dependent manner consistent with the increased secretion of beta human chorionic gonadotrophin (β-HCG) and appearance of multi-nucleated cells. Analyses of the fusion process demonstrated that in addition to increased cAMP levels, simultaneous reduction of intracellular calcium and inhibition of Type 1 phosphatidylinositol 3 kinase (PI3K)/Akt signaling also resulted in cell fusion. Although individual blockade of calcium channel function or PI3K/Akt signaling was without effect, the combination with forskolin resulted in a potentiation of cell fusion. These data demonstrate syncytialization is a complex process that depends upon the regulation of distinct signaling inputs that function in concert with each other.

## Introduction

Cellular fusion to form multi-nucleated structures is a highly coordinated process that allows tissues to develop specific properties and functions that are not possible in mononuclear cells. For example, the fusion of myoblasts to form large multi-nucleate syncytia is necessary for skeletal muscle development and for these tissues to undergo regulated contraction for force generation [1], [2]. The formation and maintenance of the placental syncytiotrophoblast structure through fusion of trophoblasts is essential for separating fetal and maternal blood and is required for successful embryonic implantation, subsequent oxygen/nutrient transport and the secretion of specific placentally derived hormones necessary for fetal development [3]. Following fertilization and prior to blastocyst formation, trophoblasts undergoing fusion form an early syncytiotrophoblast at the embryonic pole and subsequent to implantation continues to fuse with mononuclear cytotrophoblasts to generate a barrier for maternal-fetal exchange and for placental endocrine hormone secretion [4].

In general, cell-cell fusion initiates with recognition and adhesion between two potentially fusing cells followed by fusogenic pore formation, expansion and mixing of the cell surface bilayers resulting in cytoplasmic continuity [5]. A variety of studies have suggested the involvement of several proteins that are thought to be involved in the fusion process. For example, syncytin 1 and 2 are primarily syncytiotrophoblast proteins thought to interact with neutral amino acid receptors, ASCT1 or 2 that function as syncytin receptors [6], [7]. Evidence for a direct role of syncytin 1 in trophoblast cell-cell fusion is supported by anti-sense oligonucleotide treatment of primary isolated cytotrophoblasts that results in decreased cell fusion [8]. However using villus explants, syncytin 1 anti-sense oligonucleotides were also found to decrease the number of cytotrophoblast opposite to that expected if cytotrophoblast fusion was inhibited [8]. CD98 is a cell surface antigen that is expressed in cytotrophoblast and has been implicated in virus-induced cell fusion and osteoclast formation [9]. Reduction in CD98 expression by both anti-sense oligonucleotides and RNAi was also observed to inhibit cell fusion [10]. Other studies have reported a necessary role for the ADAM proteins particularly ADAM12 [11] as well as exposure of phosphatidylserine to the outer membrane leaflet have also been implicated [6], [12].

In addition to the uncertainty of the fusogenic effectors and mechanism of action, the molecular signaling pathways that regulate trophoblast fusion have also not been extensively investigated. Various studies have reported the requirement of caspase 8 activation, microtubule associated protein stathmin, transcription factor GCMa and reduction of protein tyrosine phosphatase activity implicating increased tyrosine kinase activity in the fusion process (reviewed in [6]). Despite these studies, a detailed understanding of the specific regulatory events control trophoblast fusion is critical as the integrity of the syncytiotrophoblast depends upon continuous fusion with mononuclear cytotrophoblasts [13]. To address this issue, we developed a quantitative live cell-cell fusion assay using the human choriocarcinoma BeWo cell line. This assay builds upon previous work utilizing flow cytometry [14] and use of fluorescence to separate endomitosis from fusion [15]. Using this assay system we now demonstrate that cell fusion is controlled by the regulation of calcium influx and Akt activity.

## Results

### Fusogenic characteristics of stable GFP and dsRFP expressing BeWo cells

**Figure 1:**
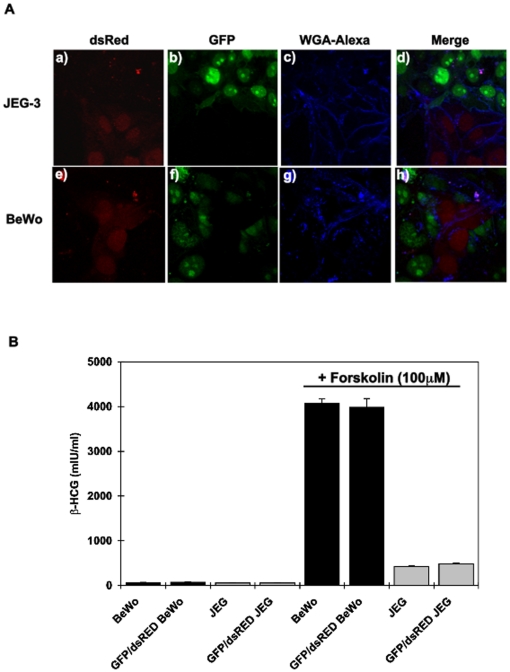
BeWo and JEG cells expressing dsRED and GFP are functionally unchanged. **A**) Fusogenic (BeWo) and non-fusogenic (JEG-3) human choriocarcinoma cell lines stably expressing GFP and dsRED each, were co-incubated and stimulated with 100 µM forskolin for 24 h. The cells were then fixed, labeled with wheat germ agglutinin (WGA) alexa. The cells were visualized by confocal fluorescence microscopy for GFP (panels b and f), dsRed (panels a and e), WGA-Alexa (panels c and g) and the merged image (panels d and h). **B**) Control BeWo (black bars) and JEG-3 (grey bars) cells as well as transfected cells were treated with vehicle or with 100 µM Forskolin for 24 hours. Incubation media were collected and β-HCG released in the media was measured using an ELISA assay. Data are expressed in mean ± s.e.m and are representative of 3 independent experiments. Stimulation with Forskolin resulted in a significant increase of β-HCG in BeWo cells *p<0.001*. A smaller but still significant increase was exhibited by JEG cells *p<0.05*. No significant difference in β-HCG release was noted between either the transfected BeWo or JEG as compared to their controls.

To develop an efficient and simple quantitative cell fusion assay, we transfected fusogenic BeWo cells and control non-fusogenic JEG-3 cells with both Green Fluorescent Protein (GFP) and Red Fluorescent Protein (dsRed) and generated stable cell lines (Fig. 1A). Mixing the BeWo-GFP and BeWo-dsRed together in the presence of forskolin (Fig.1A, panels e, f, g, h), an established activator of cAMP and inducer of BeWo cell fusion, resulted in the formation of fused cells as visualized by the presence of multiple nuclei in larger wheat germ agglutinin (WGA) labeled aggregated cells (Fig.1A, panel h). In contrast, cAMP was ineffective in the induction of JEG-3-GFP and JEG-3-dsRed cell fusion (Fig.1A, panels a, b, c, d). Confirmation that GFP and dsRed BeWo cells underwent functional membrane fusion similar to wild type BeWo cells there was a marked induction (∼1,000-fold) of β-HCG secretion following forskolin treatment that was essentially identical in wild type BeWo and the GFP/dsRed expressing BeWo cells (Fig. 1B). Furthermore, neither BeWo nor the JEG-3 cells in the basal state or the JEG-3 cells in the presence of forskolin displayed any significant levels of β-HCG secretion.

**Figure 2:**
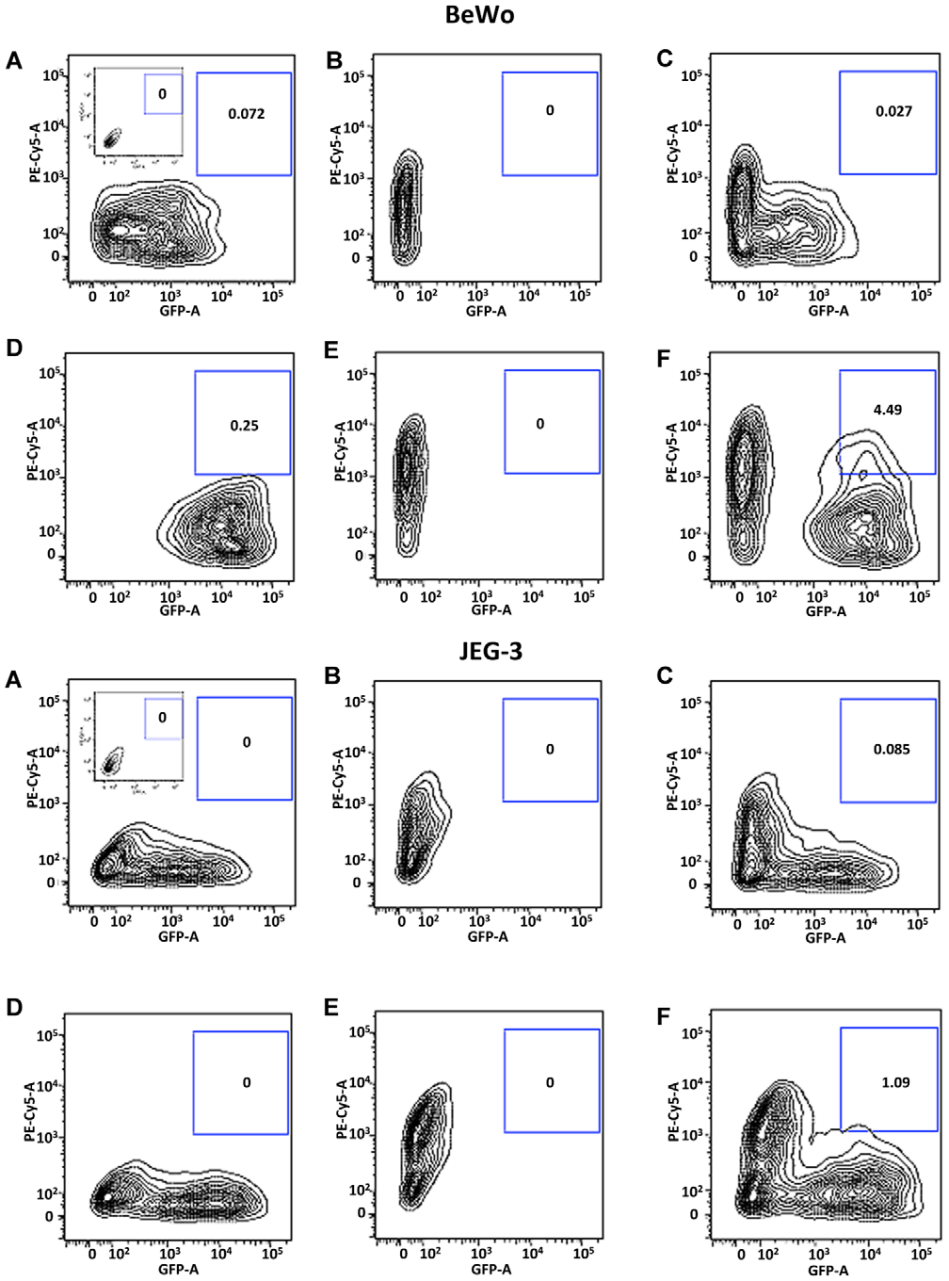
Comparison of fusion measurement utilizing flow cytometry in GFP and dsRED expressing BeWo and JEG cells. Cells were cultured with vehicle (panels **A–C**) or 30 µM forskolin (panels **D–E**) for 24 h. The results are two-parameter plots GFP fluorescence (measured on the GFP channel) or dsRED fluorescence (measured on the PE Cy-5 channel). Results were analyzed using FlowJo (8.8.6) software. Gating was performed as described in Experimental Procedures and is indicated by the boxed region on the right of each panel. BeWo cells are shown on the top panels (**A–F**) and JEG-3 cells on the bottom panels (**A–F**). Negative controls for BeWo (A, inset top left) and JEG-3 (**A**, inset bottom left) cells non-expressing GFP or dsRed. The same gates were used throughout this study and each contour map represents the counting of 10,000 events.

To establish a quantitative assay for BeWo cell fusion, we took advantage of flow cytometry to distinguish between cells that are single labeled with either GFP or dsRed versus those that are double labeled with both GFP and dsRed. As shown in contour plot in Figure 2, the majority of GFP-labeled BeWo cells in the absence of forskolin were positive for GFP with very low levels in the gated region of double positive fluorescent signal indicated by the insert box on the right (Fig. 2A, Top). The inset box on the left shows the fluorescent signals observed in control non-GFP/dsRed expressing BeWo cells. Similarly, dsRed labeled BeWo cells were only positive for Red fluorescence (Fig. 2B, Top). When the GFP-labeled and dsRed labeled BeWo cells were combined only 0.027% of the cells were scored positive for both labels (Fig. 2C, Top). Forskolin treatment of GFP labeled BeWo cells resulted in a rightward shift of the GFP signal (Fig. 2D, Top) indicating increased fluorescence per unit cell. Similarly, forskolin treatment of dsRED labeled BeWo cells resulted in an upward dsRED signal (Fig. 2E, Top) indicating increased fluorescence per unit cell. However, when the GFP and dsRed labeled BeWo cells are mixed and treated with forskolin, the individual shifts are observed along with the formation of a new population of double positive cells that increases to approximately 4.5% (Fig. 2F, Top). In contrast, GFP and dsRED JEG-3 cells individually showed a limited shift in fluorescence signal following forskolin treatment (Fig. 2A–C, Bottom) and very few double positive cells when mixed in the presence of the forskolin (Fig. 2D–F, Bottom). As a control, the inset box on the left in Figure 2A, bottom shows the fluorescent signals observed in non-GFP/dsRed expressing JEG-3 cells.

**Figure 3:**
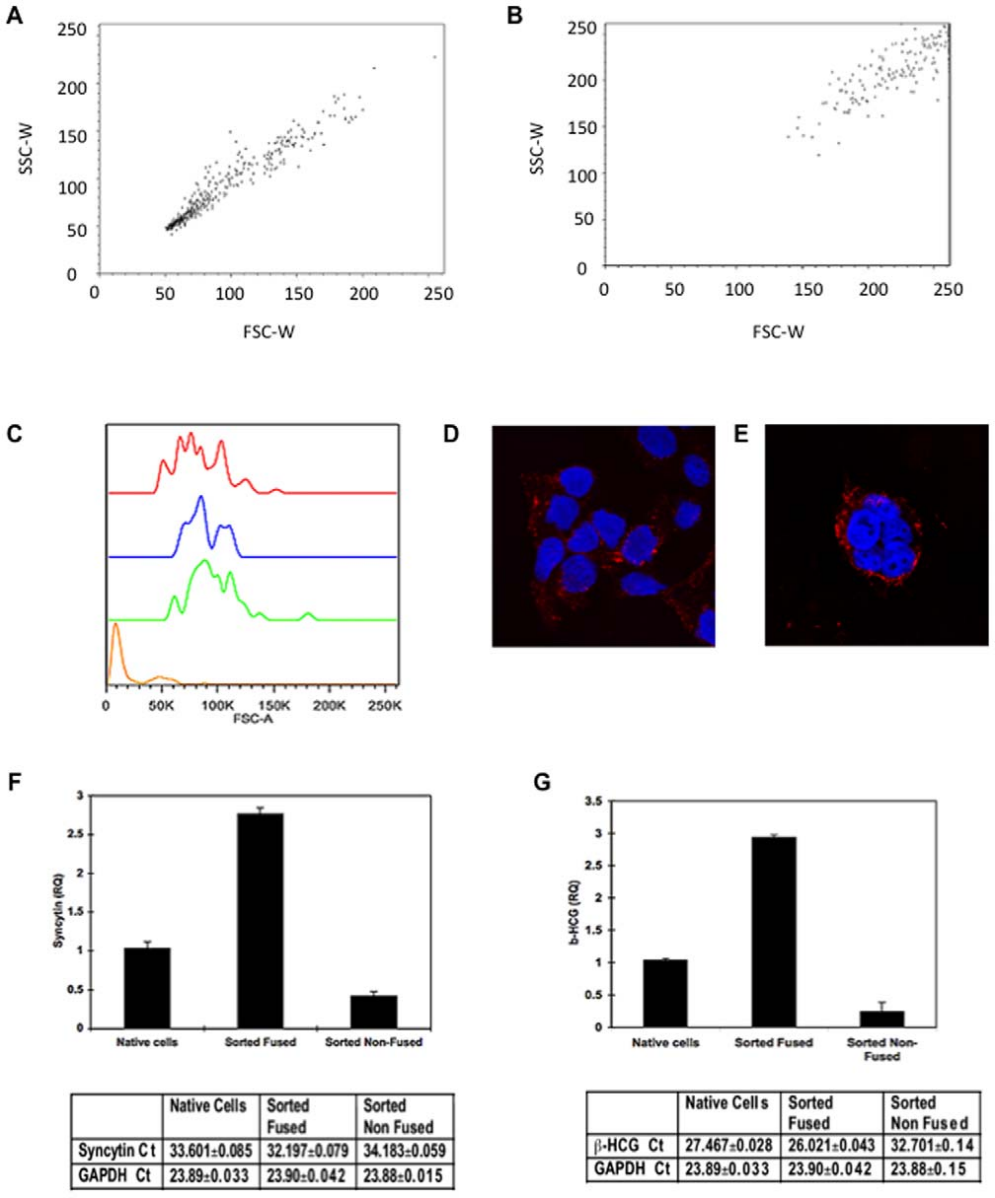
Forskolin Induces BeWo cell fusion and not aggregation. Forward-scatter width (FSC-W) was plotted against Side-scatter width (SSC-W) in unstimulated (**A**) and forskolin-stimulated (**B**) BeWo cells. Analysis of fused cells size compared to control cells (**C**). Control (orange) cells are smaller than dsRED/dsRED fused BeWo cells (red), GFP/GFP fused BeWo Cells (green) and dsRED/GFP fused BeWo cells (blue) indicating the increase in intensity is as a result of increased fusion. Representative confocal microscopic images of sorted unfused BeWo (**D**) and fused BeWo (**E**) cells. BeWo cells were incubated with vehicle or 30 µM Forskolin for 24 h. The fused and non-fused cells were sorted by FACS, RNA was extracted the expression of syncytin-1 (**F**) and ß-HCG (**G**) mRNA was quantified by quantitative RT-PCR. Relative quantitation was performed using cells treated or not with forskolin in six well plates as control. Actual Ct values are displayed underneath. Data are expressed in mean ± s.e.m and are representative of 4 independent experiments.

The forskolin-induced increase in individual GFP/dsRed fluorescence signal and double positive signal of mixed GFP and dsRed labeled BeWo cells could have resulted from either fusion of mononuclear cells into multi-nucleated cells or due to aggregation independent of fusion. To address this issue, we compared Side-scatter width (SSC-W) versus Forward-scatter width (FSC-W) by FACS analysis. The linear increase between untreated and forskolin-stimulated double BeWo cells is consistent cell fusion and not aggregation (Fig. 3A and B) [16]. Additional analysis of Forward-scatter area (FSC-A) revealed that the increased intensities seen in both dsRED and GFP BeWo cells treated with forskolin represented fusion of these cell populations (Fig. 3C) since their size was commensurate with those of the double positive cells, as previously been reported [17]. Confocal microscopy confirmed that the double positive BeWo cells were mainly composed of fused cells characterized by a multinucleated syncytium (Fig. 3E). In contrast, in absence of forskolin, the cell population contained mono-nucleated individual cells that displayed no change in cell size (Fig. 3D). In addition, quantitative-PCR (q-PCR) analysis revealed that expression of fusion markers such as syncytin-1 and β-HCG was increased in the sorted fused cells, consistent with the fused BeWo cell population displaying normal syncytiotrophoblast function (Fig. 3F and G).

**Figure 4:**
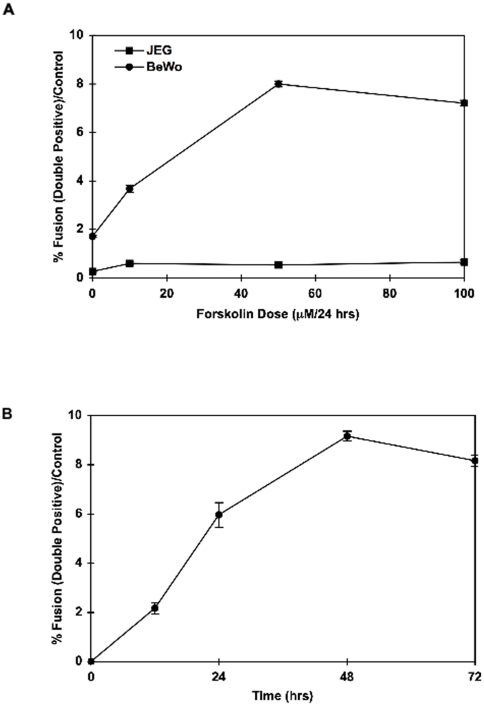
Dose and Time Response of BeWo fusion. **A**) BeWo (circles) and JEG cells (squares) were incubated with the indicated concentrations of forskolin for 24 h and the proportion of fused cells was analyzed by flow cytometry. **B**) BeWo cells were incubated with 30 µM forskolin for the times indicated and the proportion of fused cells was determined by flow cytometry at the indicated time points. Data are expressed in mean ± s.e.m and are representative of 4 independent experiments.

Having established a quantitative fusion assay, we next optimized syncytialization by examining the time and dose-responsiveness of forskolin stimulation cell fusion. As shown in Figure 4A, following 24 h of stimulation maximum fusion of BeWo cells occurred at 50 µM forskolin. In contrast, the non-fusogenic JEG-3 cells remained unresponsive and cellular fusion was not significant even with 100 µM forskolin treatment. In addition, syncytialization of the BeWo cells was readily detectable as early as 12 h after forskolin treatment (50 µM) with maximum fusion at 48 h (Fig. 4B). Therefore, having optimized conditions for cell fusion, in all subsequent studies we stimulated BeWo cells with 30 µM forskolin for 24 h since this was on the linear part of the forskolin dose response and time course curves.

### BeWo cell fusion is enhanced by LY294002 but unaffected by wortmannin

**Figure 5:**
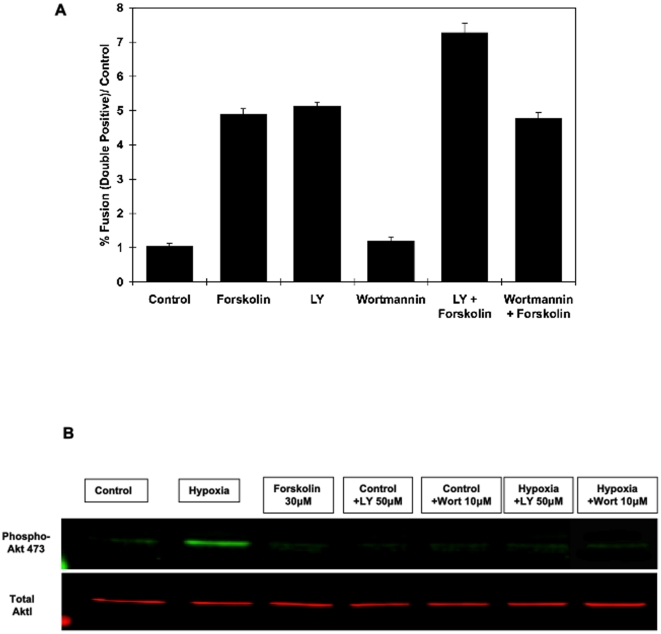
Differential effects of LY294002 and wortmannin on fusion. BeWo cells (**A**) were incubated with 50 µM LY294002 (LY) or wortmannin (10 µM) alone or with forskolin (30 µM) for 24 h and the proportion of fused cells was determined by flow cytometry analysis. LY294002 and wortmannin are effective inhibitors of PI3 kinase at the concentrations used (**B**). Total Akt and pS473-Akt was immunoblotted in BeWo cell extracts from vehicle treated control, hypoxia-stimulated, 30 µM forskolin-stimulated, 50 µM LY, 10 µM wortmannin, hypoxia plus LY and hypoxia plus wortmannin treated cells. This is a representative immunoblot independently performed 4 times.

The Type 1 phosphatidylinositol 3-kinase (PI3K) is a central signaling node for many extracellular effectors that integrates a variety of intracellular biological responses [18]. We therefore initially examined the potential involvement of Type 1 PI3K-dependent signals in the control of BeWo cell fusion. As typically observed, in the absence of forskolin the reporter BeWo cells displayed a relatively low level of fusion that was increased approximately five-fold following forskolin treatment (Fig. 5A). Treatment with the PI3K inhibitor LY294002 (LY) also resulted in an equivalent increase in cell fusion and potentiated the extent of fusion in the presence of forskolin. Surprisingly however, the PI3K inhibitor wortmannin was completely without effect and was unable to potentiate cell fusion induced by forskolin. Both LY and wortmannin were able to inhibit phospho-Akt, which is downstream of PI3K, at the concentrations used (Fig. 5B). Since LY294002 is a less specific PI3K inhibitor than wortmannin, these data suggest that LY294002 was having an effect through another pathway in conjunction with or independent of PI3K function.

**Figure 6:**
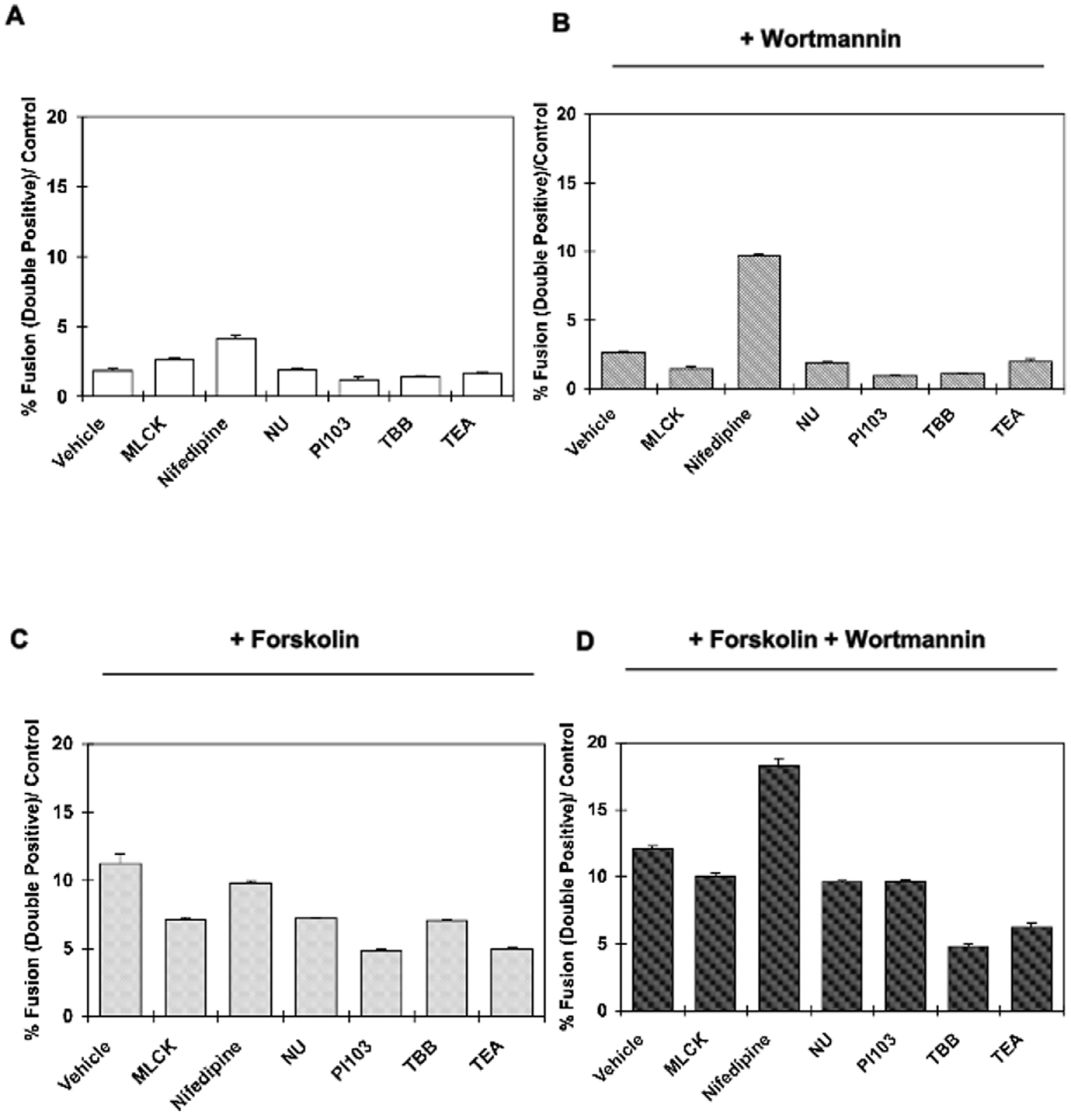
Dissection of LY294002 effects on fusion. BeWo cells were incubated for 24 h with different inhibitors (described in the manuscript) alone (**A**) or in presence of 10 µM wortmannin (**B**) and proportion of fused cells was assessed by FACS analysis. Nifedipine (10 µM) significantly increased fusion in the presence of 10 µM wortmannin (*p<0.001)*. The effects of 30 µM forskolin was determined with the different inhibitors alone (**C**) or in presence of 10 µM wortmannin (**D**) and proportion of fused cells was assessed by FACS analysis. Nifedipine significantly increased fusion when 30 µM forskolin and 10 µM wortmannin were used together. (*p<0.001*). Data are expressed in mean ± s.e.m and are representative of 3 independent experiments.

Therefore, we next screened for other known targets of LY294002 to regulate BeWo cell fusion. As shown in Figure 6A, treatment of BeWo cells with inhibitors of the myosin-light chain protein kinase, peptide 18 (MLCK), the DNA-dependent protein kinase inhibitor NU1076 (NU), the broad inhibitor of the PI3K family PI103, the inhibitor of creatine kinase-2 (4,5,6,7-tetrabromobenzotriazole; (TBB)), and the potassium channel inhibitor tetraethylammonium (TEA) all had no effect on BeWo cell fusion, whereas the calcium channel inhibitor Nifedipine appeared to have small effect to increase cell fusion. However, in the presence of wortmannin, to inhibit the Type 1 PI3K, the co-inhibition of calcium channel function by nifedipine resulted in a substantial increase in BeWo cell fusion (Fig. 6B). Although several of the tested inhibitors may have small effects to reduce the forskolin stimulation of BeWo cell fusion, importantly nifedipine (like wortmannin alone) did not enhance forskolin-stimulated fusion (Fig. 6C). However, the combination of nifedipine and wortmannin together increased the effects of forskolin on BeWo cell fusion, reproducing the effects of the LY294002 inhibitor (Fig. 6D). These data strongly suggest that the positive effects of the LY294002 on BeWo fusion are a result of PI3K inhibition in combination with the inhibition of the calcium channel activity. Additionally, both pathways are necessary and function concurrently in the syncytialization process, as wortmannin and nifedipine alone do not display any significant effects on forskolin-induced BeWo cell fusion.

### BeWo fusion requires both a decrease of intracellular calcium concentration and Akt inhibition

**Figure 7:**
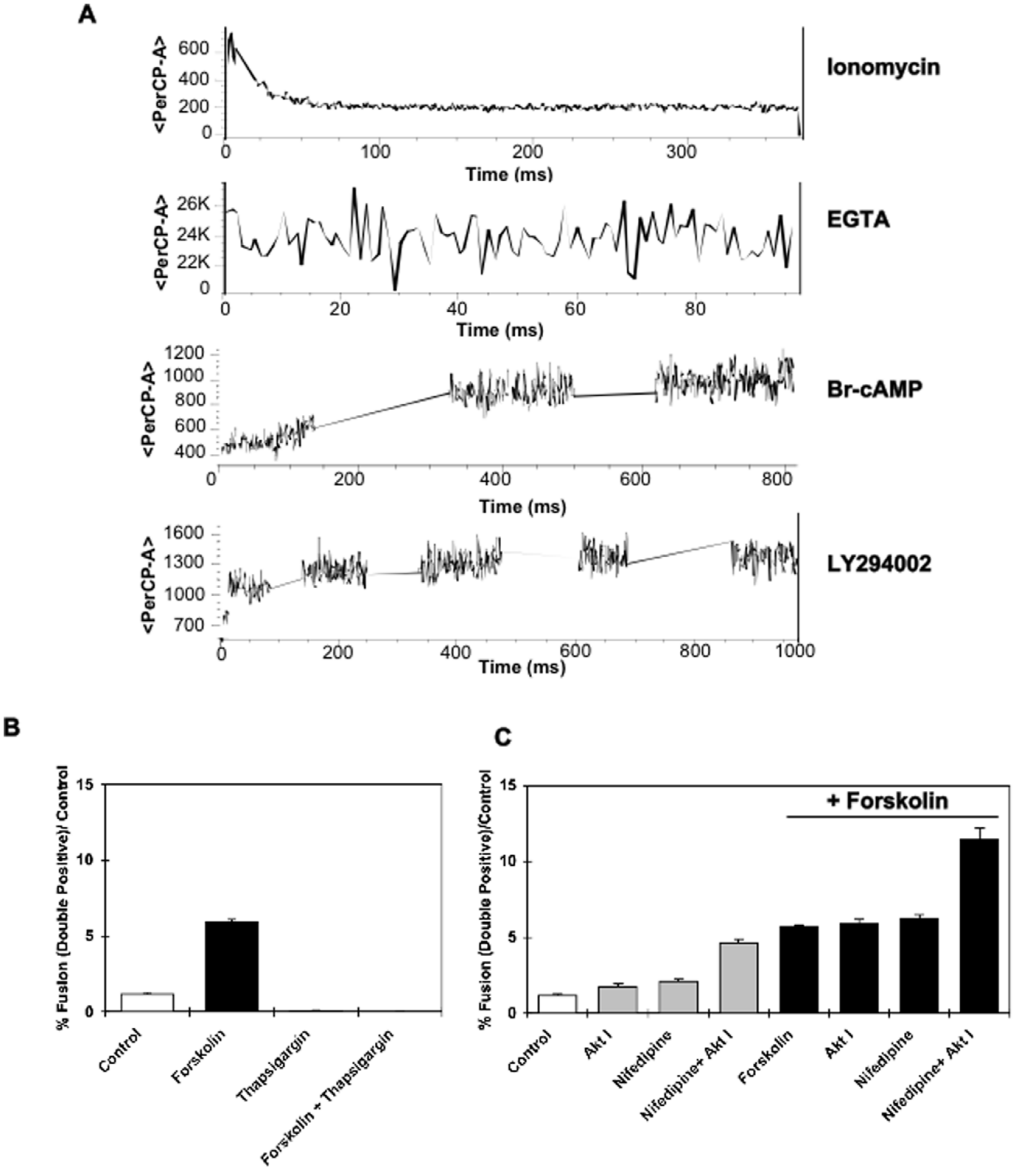
The role of calcium in BeWo syncytialization. **A**) Fura red was used as an inverse intracellular calcium indicator and calcium transients were measured in real time utilizing Flow Cytometry. Fura Red was measured with the perCP channel at 488 nm. The top panel shows the effects of ionomycin 1 µM on the calcium transient with a decrease in intensity as intracellular calcium increases. The second panel shows the increase in fura red as calcium levels are decreased by EGTA. In the third and fourth panels, real time decreases in intracellular calcium are seen with both addition of cAMP (8-bromo-cAMP 1.5 mM - third panel) and LY294002 (50 µM - fourth panel). **B**) Effects of thapsigargin (100 nM) on BeWo fusion were determined by incubating the BeWo cells with thapsigargin in the presence or absence of 30 µM forskolin for 24 h. Thapsigargin completely abolished the presence of double positivity assessed by flow cytometry. C) Cells were incubated with vehicle (control, open bar) or with 30 µM forskolin for 24 hr (black bars). The incubations were repeated in the presence of nifedipine (10 µM) or in the presence of nifedipine (10 µM) and Akt Inhibitor VIII (Akt I at 10 µM). Nifedipine alone did not influence fusion, but nifedipine and Akt Inhibitor VIII increased fusion in both control and forskolin treated cells.

If the calcium channel is involved in BeWo cell fusion, then intracellular calcium levels should be changed during this process. To address this issue, intracellular calcium levels during the syncytialization process was determined using flow cytometry in native BeWo cells labeled with the inverse calcium indicator fura red (Fig. 7A). Addition of 8-Bromo-cAMP resulted in a marked time-dependent decrease in intracellular calcium levels. Similarly, LY294002 also elicited a decrease in intracellular calcium levels. To examine the role of intracellular calcium influx regulating BeWo cell fusion, we took advantage of thapsigargin, a sesquiterpene lactone that raises cytosolic calcium concentration by blocking the calcium pump of the endoplasmic reticulum [19]. Thapsigargin completely abrogated forskolin-induced fusion of the BeWo (Fig. 7B). Together these data demonstrate that the syncytialization process is dependent on a decrease in intracellular calcium concentration, consistent with the effects of nifedipine.

Since Akt is a major downstream target for the Type 1 PI3K signaling pathway, we determined the role of Akt in concert with calcium channel inhibition. Treatment with forskolin resulted in the typical induction of BeWo cell fusion (Fig. 7C). Blockade of Akt activation using the Akt1/2 inhibitor (Akt I) alone or in combination with forskolin had no additional affect. As previously observed, calcium channel inhibition (nifedipine) alone or in combination with forskolin also had no additional effect. However, both nifedipine and Akt inhibitor treatment mirrored the effect of LY294002 effectively stimulating BeWo cell fusion and potentiating the fusion in the presence of forskolin. These data demonstrate that maximum BeWo cell fusion is a complex process that requires increased intracellular cAMP coupled with decreased intracellular calcium levels and inhibition of PI3K/Akt signaling.

## Discussion

The multi-nucleated syncytiotrophoblast provides the interface that separates fetal and maternal blood and functions in the transport of various nutrients, metabolic waste products and synthesis of a variety of essential hormones [20]. The syncytiotrophoblast is generated and maintained by the fusion with mononuclear cytotrophoblasts, as disruption of cytotrophoblast fusion results in syncytiotrophoblast necrosis [21]. Understanding the complex cell biological and signaling processes responsible for regulating cytotrophoblasts fusion has become an emerging issue as alterations in this process are associated with preeclampsia, a clinical condition associated with hypertension and proteinuria that affects as many as 10% of all pregnancies [22].

Currently, several groups have collectively identified a variety of potential targets involved in the fusion process. However, we still have a rudimentary knowledge of the physiologically relevant fusion proteins, their mechanisms of action and signaling pathways that control these processes. One barrier to advances in this field has been the lack of a readily simple and quantifiable assay for cytotrophoblast fusion. To address this need, we have developed a sensitive and highly reproducible system taking advantage of the human chorinocarcinoma cell line BeWo, used as a representative *in vitro* system for cytotrophoblast cell-cell fusion. By generating stable GFP and dsRed BeWo cell lines, these reporter cells can be mixed and, following standard stimulation via elevation of intracellular cAMP fusion can be readily quantified using fluorescent activated cell sorting. In this system, treatment with forskolin induces a robust time and concentration increase in cell-cell fusion from a low non-stimulated background. However, since each cell whether mono or multi-nucleated is counted as one unit, this assay actually underestimated the total extent of fusion. The average number of nuclei per *in vitro* syncytium under these conditions is 6 (data not shown), thus the average number of nuclear fusion events following forskolin stimulation is in actuality approximately 22%. In any case, these data demonstrate that the fusion of the stable fluorescent protein-tagged BeWo cells provides a very sensitive, efficient and reproducible cell-cell fusion assay system. Moreover, the failure of the non-fusogenic cell line JEG-3 to display any significant double positivity reassured us that the phenomena we were measuring represented cell fusion as opposed to cell aggregation. The additional advantage of this assay was that it utilizes live cells permitting the downstream interrogation of these cells using either hormonal assays or quantitative PCR.

Using this system we have uncovered an unexpected interplay between intracellular calcium and the PI3K/Akt signaling pathway that appears to function in parallel with the cAMP pathway mediating cell fusion. Although forskolin elevation of cAMP is an effective inducer of BeWo cell fusion, this was completely blocked by increasing intracellular calcium levels. Moreover, 8-Bromo-cAMP also resulted in a reduction in intracellular calcium. The requirement for reduced intracellular calcium in BeWo cell fusion is quite surprising as myoblast fusion is driven by increased intracellular calcium and highlights tissue specific differences in these fusion mechanisms [23]. Although inhibition of calcium influx appears to be functionally important in BeWo cell fusion [24], it is not sufficient as nifedipine itself does neither induce cell fusion nor potentiates forskolin-mediated fusion.

In concert with calcium channel inhibition, inhibition of PI3K/Akt signaling itself was also not sufficient to induce BeWo cell fusion and also was unable to potentiate forskolin-stimulated fusion. However, the combination of calcium channel inhibition with PI3K/Akt inhibition was just as effective as forskolin in stimulating BeWo cell fusion. Furthermore, calcium channel and PI3K/Akt inhibition together potentiated forskolin-stimulated cell fusion. Thus these finding underscore that multiple signaling pathways can functional cooperate to drive syncytialization and suggest these signals allow for precise regulation of placental development in vivo based upon the maternal/fetal environment. With this live cell assay system in-hand, further studies can now be initiated to determine the upstream signals and downstream targets that functionally mediate trophoblast cell-cell fusion.

## Methods

### Cells and culture conditions

Fusogenic BeWo cells and non-fusogenic JEG-3 cells were obtained from ATCC (Manassas, VA, USA) and cultured in F-12K (Invitrogen, Carlsbad, CA, USA) or Dulbecco's Modified Essential Medium (DMEM, Invitrogen, Carlsbad, CA) respectively, both supplemented with 10% heat inactivated fetal bovine serum (Invitrogen, Carlsbad, CA, USA) and 100 units/ml penicillin, and 100 mg/ml of streptomycin (SigmaAldrich, St. Louis, MO, USA).

### Lentiviral packaging

pLVX-DsRed-Monomer (Clontech CA, USA) was packaged as lentivirus in HEK-293 cells using lentivirus packaging mix (SigmaAldrich, St. Louis, MO, USA) according to the manufacturer's instructions. Briefly, HEK293T cells in DMEM, supplemented with 10% fetal bovine serum and 4 mM L-glutamine, were grown to 70%–80% confluence in 100 mm dishes. On the second day, 182 µl of OPTI-MEM serum free media and 16 µl of FUGENE 6 (Roche, IN, USA) transfection reagent were combined with a transfection cocktail of 26 µl of lentiviral transfection mix and 2.6 µg of pLVX-DsRed-Monomer and added to the cells. The subsequent day, the cells were fed with 10 ml of DMEM and after 16 h this media was removed and replaced with fresh media. The cells were incubated for a further 36 h when the media were collected and stored at 4°C. Media were replaced and collected again 24 h later. After filtering the collected medium through 0.45 µm filters, the virus was concentrated by spinning at 4,000 g for 15 min followed by a second spin (1,000 g for 2 min at room temperature). The concentrated virus was stored at -80°C. The titer of lentiviral vectors was determined by dilution. GFP lentiviral transduction particles were purchased ready made from Sigma (SHC003 at 10^9^ TU/ml).

### Lentiviral Transduction

Lentiviral transduction of BeWo and JEG-3 cells was performed according to the manufacturer's protocol (SigmaAldrich, St. Louis, MO, USA). Briefly, 1.6×10^4^ of BeWo and JEG-3 cells were added to wells (in triplicate for GFP and dsRED) in a 96 well plate and incubated for 20 h to achieve a confluency of 70%. The media were removed and replaced with 110 µl Minimum Essential Medium (MEM) supplemented with 10% Fetal calf serum and hexadimethrine bromide (final concentration 8 µg/ml, SIGMA) containing 5 µl of GFP virus or 12 µl of the dsRED virus. The following day, the media containing virus were removed and 120 µl of fresh media were added. On day 3, the media was removed and replaced with fresh media containing puromycin at 6 µg/ml. This was continued until colonies expressing GFP or dsRED were identified. These were then expanded. dsRED virus was calculated as 10^8^ TU/ml. The MOI for GFP was 4 in both BeWo and JEG-3, the MOI for dsRED was 8 in both cell types.

### Beta-HCG quantification

Assessment of beta-HCG release by control and lentiviral transduced BeWO and JEG-3 cells was assessed by addition of 50 µM forskolin (EMD Biosciences, Gibbstown, NJ, USA) for 24 h. The beta-HCG released in the media was determined using an ELISA kit from R&D (Minneapolis, MN, USA) according to the manufacturer's instructions.

### Flow cytometry

After the treatments, cells were trypsinized and suspended in HBSS containing 2% Fetal calf serum and 0.2 mg/ml DNAse and filtered through a 100 micron filter to minimize aggregation. These cells were placed on ice and immediately processed by flow cytometry.

Flow cytometry was performed on GFP and dsRed expressing BeWo cell lines using BDFACSCORP ARIA equipped with Blue (488 nm), Green (532 nm), Yellow (561 nm), Red (638 nm) and Violet lasers (407 nm). The blue and the Yellow-green lasers were used to excite GFP and dsRED, respectively. 548/20 nm band pass filter was used for collecting GFP signals, and a 660/20 nm band pass filter was used to collect dsRed signals. Gating strategy: A total of 10 000 events were collected for analysis. Results were analyzed using FlowJo (8.8.6) software. Briefly, Side-scatter height (SSC-H) was plotted against Side-scatter width (SSC-W) to exclude doublets and identify live cells; the cells were then subjected to analysis with Forward-scatter height (FSC-H) against Forward-scatter width (FSC-W) to ensure doublet exclusion. Subsequently, GFP/dsREd single or double positive populations were quantified. Analysis of double positive cells revealed a linear increase in size compared to control cells confirming doublet exclusion (Figure S1).

### Western blotting

Cells were rinsed three times with PBS then lysed with ice-cold lysis buffer [50 mM Tris-HCl (pH 7.5), 0.1% (vol/vol) Triton X-100, 1 mM EDTA, 1 mM EGTA, 50 mM NaF, 10 mM sodium-glycerophosphate, 5 mM sodium pyrophosphate, 1 mM sodium vanadate, 0.1% (vol/vol) 2-mercaptoethanol, and protease inhibitors (1:1000 dilution of protease inhibitor cocktail; Sigma)]. Lysate protein content was determined using the Bio-Rad Bradford procedure. Proteins were resolved by SDS-PAGE, transferred to PVDF membrane and subjected to immunoblotting with specific antibodies (phospho-Akt & Total Akt (Cell Signaling, Danvers, MA, USA). Proteins were visualized with the Odyssey Infrared Imaging System (LI-COR Biosciences, Lincoln, NE, USA). Induction of Akt phosphorylation, as a measure of Akt activation, was accomplished by incubating the BeWo cells in hypoxic conditions as previously described [25].

### Intracellular calcium determination

BeWo cells were labeled with Fura Red AM (Invitrogen, Carlsbad, CA, USA), an inverse fluorescent calcium probe which decreases in intensity upon calcium binding. Cells were labeled by incubating cells suspended in 1.5 ml buffer (HBSS containing 2% Fetal calf serum and 0.2 mg/ml DNAse) with Fura Red AM at a final concentration of 10 µM for 20 min at 37°C and washing the cells with PBS prior to experiments. cAMP (8-bromo-cAMP 1.5 mM) and LY294002 (50 µM) were added immediately prior to processing via flow cytometry. For calcium free experiments, cells were suspended in buffer containing EGTA (10 µM). Ionomycin 3 µM was added just prior to FACS analysis for investigation of raised intracellular calcium. Measurements of calcium transients were performed using BDFACSCORP ARIA with excitation at 488 nm and emission at 590 nm.

### Inhibitors

Nifedipine (Calcium channel inhibitor) was used at a final concentration of 10 µM and tetraethylammonium chloride (potassium channel inhibitor) was used at a final concentration of 5 mM (both SigmaAldrich, St. Louis, MO, USA). NU7026 (DNA dependent protein kinase inhibitor) was used at a final concentration 0.3 µM, TBB (4,5,6,7-Tetrabromobenzotriazole, a casein kinase 2 inhibitor) at a final concentration of 10 µM, PI103 (3-[4-(4-Morpholinylpyrido[3′,2′:4,5]furo[3,2-d]pyrimidin-2-yl]phenol hydrochloride, an inhibitor of mTOR and p110alpha) was used at a final concentration of 1 µM , MLCK Peptide 18 (Myosin Light Chain Kinase Inhibitor Peptide 18) was used at a final concentration of 1 µM (all from Tocris Ellisville, MO, USA). Akt Inhibitor VIII, Isozyme-Selective was used at a final concentration of 10 µM (EMD Chemicals Gibbstown, NJ, USA). The selection of inhibitor concentrations used was based on manufacturer's data and comparable published studies.

### Confocal Microscopy

Fused and non-fused cells were sorted and collected by flow cytometry and placed on Lab-Tek chamber slides (Nalge Nunc International, Rochester, NY). The slides were fixed and cell membranes were stained with wheatgerm agglutinin conjugated to Alexa Fluor 594 (SigmaAldrich, St. Louis, MO, USA) whilst nuclei were stained using ProLong antifade with DAPI (Invitrogen, Carlsbad, CA, USA). Slides were visualized by confocal fluorescence microscopy using a TCS SP5 laser scanning confocal microscope (Leica microsystems, Buffalo Grove, IL, USA).

### Quantitative PCR analysis

Fused or non-fused cells were collected either from cell sorting or from wells (as controls), immediately placed into QIAzol Lysis Reagent (Quiagen, Valencia, CA, USA) according to the manufacturer's protocol. Total RNA was isolated using RNeasy® Mini Kit (Qiagen Sciences, Maryland, USA) and then reverse-transcribed to cDNA using the SuperScript VILO cDNA synthesis kit (Invitrogen, Carlsbad, CA, USA). TaqMan (Applied Biosystems, Branchburg, NJ, USA) RT-PCR was performed for measurement of mRNA for fusion markers beta-HCG and syncytin. Relative expression levels of the mRNAs were determined using standard curves. Samples were adjusted for total mRNA content by comparison with GAPDH expression. All primer-probe mixtures were from Applied Biosystems (Branchburg, NJ, USA).

### Statistics

Results are expressed as mean ± standard error of the mean (s.e.m). Differences between incubation conditions were tested for statistical significance (*p<0.05*) using Student's unpaired t-test. All experimental treatments were performed in biological triplicate with each biological replicate having technical triplicates.

## Supporting Information

Figure S1 **Forskolin-stimulated BeWo cell fusion and not aggregation.** **A**) GFP and dsRed labeled BeWo cells were mixed, treated with 30 µM forskolin for 24 h and analyzed for Side-scatter Height (SSC-H) versus Side-scatter width (SSC-W) as an assessment aggregation. **B**) This population was then analyzed for Forward-scatter Height (FSC-H) versus Forward-scatter width (FSC-W) to confirm the absence of aggregation. **C**) The BeWo cells were then assessed using PE-Cy5 (for dsRED) and GFP (for GFP). The non-fused (**D**) and fused (**E**) cells were assessed for size using Forward-Scatter Area (FSC-A) versus Side-scatter Area (SSC-A) revealing that they were smaller than the fused cells and were not aggregated as revealed by their linear increase.
(TIF)
