# Supplementary figures and images for: Inhibition of Akt Activity and Calcium Channel Function Coordinately Drive Cell-Cell Fusion in the BeWO Choriocarcinoma Placental Cell Line
Source: PLoS One. 2012 Jan 19;7(1):e29353. doi: 10.1371/journal.pone.0029353 (PMC3261872; doi:10.1371/journal.pone.0029353)

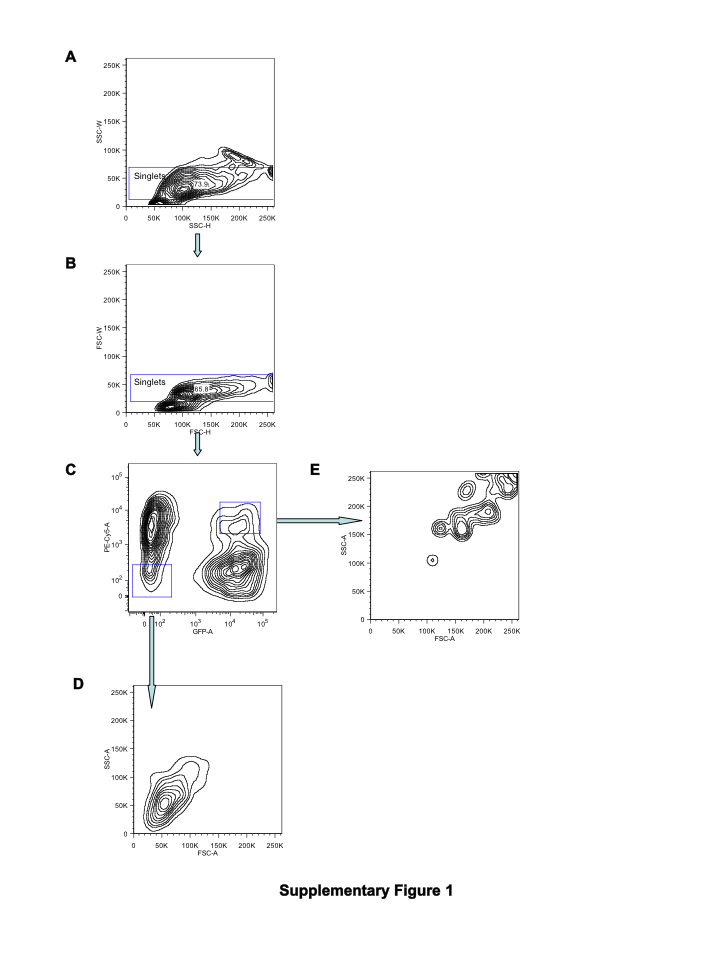

Supplement: Figure S1 — Forskolin-stimulated BeWo cell fusion and not aggregation. A) GFP and dsRed labeled BeWo cells were mixed, treated with 30 µM forskolin for 24 h and analyzed for Side-scatter Height (SSC-H) versus Side-scatter width (SSC-W) as an assessment aggregation. B) This population was then analyzed for Forward-scatter Height (FSC-H) versus Forward-scatter width (FSC-W) to confirm the absence of aggregation. C) The BeWo cells were then assessed using PE-Cy5 (for dsRED) and GFP (for GFP). The non-fused (D) and fused (E) cells were assessed for size using Forward-Scatter Area (FSC-A) versus Side-scatter Area (SSC-A) revealing that they were smaller than the fused cells and were not aggregated as revealed by their linear increase. (TIF) [file pone.0029353.s001.tif]
